# HPTLC-PCA Complementary to HRMS-PCA in the Case Study of *Arbutus unedo* Antioxidant Phenolic Profiling

**DOI:** 10.3390/foods8080294

**Published:** 2019-07-27

**Authors:** Mariateresa Maldini, Gilda D’Urso, Giordana Pagliuca, Giacomo Luigi Petretto, Marzia Foddai, Francesca Romana Gallo, Giuseppina Multari, Donatella Caruso, Paola Montoro, Giorgio Pintore

**Affiliations:** 1 Department of Chemistry and Pharmacy, University of Sassari, Via F. Muroni, 23/b, 07100 Sassari, Italy; 2 Department of Pharmacological and Biomolecular Sciences, Università degli Studi di Milano, Via Balzaretti, 9, 20133 Milan, Italy; 3 Department of Pharmacy, University of Salerno, Via Giovanni Paolo II, 84084 Fisciano (SA), Italy; 4 National Center for Drug Research and Evaluation, Viale Regina Elena, 299, 00161 Roma, Italy

**Keywords:** HPTLC, LC–HRMS, PCA, metabolomics, Arbutus unedo, antioxidant activities

## Abstract

A comparison between High-Performance Thin-Layer Chromatography (HPTLC) analysis and Liquid Chromatography High Resolution Mass Spectrometry (LC–HRMS), coupled with Principal Component Analysis (PCA) was carried out by performing a combined metabolomics study to discriminate *Arbutus unedo* (*A. unedo*) plants. For a rapid digital record of *A. unedo* extracts (leaves, yellow fruit, and red fruit collected in La Maddalena and Sassari, Sardinia), HPTLC was used. Data were then analysed by PCA with the results of the ability of this technique to discriminate samples. Similarly, extracts were acquired by non-targeted LC–HRMS followed by unsupervised PCA, and then by LC–HRMS (MS) to identify secondary metabolites involved in the differentiation of the samples. As a result, we demonstrated that HPTLC may be applied as a simple and reliable untargeted approach to rapidly discriminate extracts based on tissues and/or geographical origins, while LC–HRMS could be used to identify which metabolites are able to discriminate samples.

## 1. Introduction

Plant metabolic profiling is a very useful strategy to study the complexity and the large variety of compounds belonging to different chemical classes [1] and is ideally suited to comparing many samples in order to classify them according to botanical, geographical origins and chemotypes [2,3,4,5].

As a consequence, one of the many aims of research in the field of metabolomics is to analyze a large number of samples and obtain information in the shortest times and with a little or no sample preparation time [6,7]. Creation of rapid and convenient methods for simultaneous metabolites fingerprinting and their quantification requires the use of peculiar analytical techniques. 

In the last few years, the progress and developments of analysis techniques, including advanced hyphenated techniques (like Liquid Chromatography–Mass Spectrometry (LC–MS) and Gas Chromatography–Mass Spectrometry (GC–MS)), can effectively satisfy this demand [8,9,10].

Until now, most metabolomics studies were performed using the most popular analytical technologies, such as Nuclear Magnetic Resonance, GC, LC, and MS [11]. The new developments reached, employing high chromatographic resolution/separation interfaced with high resolution mass spectrometry, showed the power of this coupled technique in metabolomics [12,13,14,15]. High Performance Thin Layer Chromatography (HPTLC) is gaining more attraction in the field of metabolomics. In fact, recently, several papers have reported how HPTLC is an alternative method for routine analysis of complex matrices [5,16,17,18]. 

If, on one side, liquid chromatography–high resolution mass spectrometry is almost the preferred technique to perform screening studies of complex matrices, on the other hand, it requires many steps for metabolomics studies, in primis, among all, time run acquisitions, processing and normalization of raw data (baseline and phase correction, alignment), and, in some cases, a long and expensive sample preparation, too. In this context, High Performance Thin Layer Chromatography (HPTLC) has been acknowledged as a potent and suited analytical platform because it shows high accuracy, adaptability, flexibility, and reproducibility and because it is able to generate a quick fingerprint of diverse samples in a unique analysis [19]. HPTLC fingerprinting coupled with Principal Component Analysis (PCA) results in a consistent untargeted approach that is able to distinguish samples based on the tissues and their geographical origin [20,21,22].

*Arbutus unedo*, also named “strawberry tree”, is an evergreen shrub belonging to the Ericaceae family. It is distributed mainly in the Mediterranean region, in particular in Southern European countries, where it grows especially near the sea. Fruits and leaves possess a wide series of biological activities, such as astringent, depurative, diuretic, anti-inflammatory, antioxidant properties, attributable to their rich content in phenolic compounds. In addition, *Arbutus unedo* produces edible berries and covers economic importance in the sense of its use for the production of beverages, liquors, jams, marmalades, and of a unfloral honey. Sardinia is probably the largest producer in the world of this honey with a characteristic taste, also known as “bitter honey” [23,24].

The specific purposes of the present study were: (a) Compare the possibility to use HPTLC–PCA as an alternative to more comprehensive LC– Elettrospray (ESI)–Orbitrap–MS PCA approaches to preliminary discriminate samples of *A. unedo* (leaves, yellow fruit, and red fruit collected in Sassari and in the archipelago of La Maddalena). The samples were from two somewhat different habitats: Plants from Sassari grew about 15 km from the sea (220–230 m a.s.l.), while plants from La Maddalena grew in a coastal site (0–20 m a.s.l.); (b) identify secondary metabolites by LC–ESI–Orbitrap–MS and LC–ESI–Orbitrap–MS/MS and evaluate tentatively which ones contribute most to the distinction of the samples; (c) study the relationship between antioxidant activity assayed by DPPH (2,2-Diphenyl-1-picrylhydrazyl) and ABTS (2,2’-azino-bis(3-ethylbenzothiazoline-6-sulphonic acid)) and total phenolic content performed by Folin–Ciocalteu’s method.

## 2. Materials and Methods

### 2.1. Reagents and Chemicals

HPLC–MS grade methanol, acetonitrile, and formic acid were purchased from Sigma–Aldrich Chemical Company (St Louis, MO, USA). HPLC grade water (18 mΩ) was obtained by using a Milli-Q purification system, Millipore (Bedford, MA, USA). Chemicals and reagents necessary for antioxidant activity assays were supplied by Sigma (Dorset, UK).

### 2.2. Sampling Sites and Extraction

Wild plants of *A. unedo* were collected in October 2015 in two selected geographical areas of Sardinia: Sassari and La Maddalena. Botanical identity of the plants was assigned by Doctor M. Chessa. Voucher specimens were left at the Erbarium SASSA of Sassari University (number 514).

Samples were collected in triplicate, thus resulting in 18 biological samples, classified into 6 groups as in the following: LMFG (Yellow Fruit La Maddalena); LMFR (Red Fruit La Maddalena); LML (Leaves La Maddalena); SSFG (Yellow Fruit Sassari); SSFR (Red Fruit Sassari); SSL (Leaves Sassari). 

Leaves and yellow and red fruits of *A. unedo* from Sassari and La Maddalena were extracted under ultrasound agitation for 1 h, with ethanol/water (70:30 *v*/*v* using a sample to solvent ratio 1:10 *w*/*v*); then samples were stored in the dark overnight. Samples were then filtered and dried using a rotary evaporator under a vacuum and temperature of 30 °C, working in the dark. 

For qualitative analysis, dried samples were dissolved again in methanol to generate a solution of 1 mg/mL. The solutions were filtered through 0.20 µm syringe PVDF filters (Whatmann International Ltd., Maidstone, UK). 

### 2.3. HPTLC Analyses

The HPTLC analyses were performed following the protocol described by Maldini et al., 2016 [16], with slight modification. Extracts were reconstituted at a concentration of 6 mg/mL and a volume of 6 μL was loaded. The developing solution for HPTLC plates was a mixture of ethyl acetate/dichloromethane/acetic acid/formic acid/water (100:25:10:10:11; *v*/*v*/*v*/*v*/*v*). The length of the chromatogram run was 70 mm from the point of application.

For the densitometric analysis, a CAMAG TLC scanner 3 (CAMAG, Muttenz, Switzerland) linked to winCATS software (version 1.2.1, CAMAG, Muttenz, Switzerland). was set at 254 nm and 366 nm, after an optimization performed by a multi-wavelength mode from 220 to 700 nm. A minimum background compensation was performed on the *x*-axis during the scanning. Deuterium and tungsten lamps were used as sources of radiation. The slit dimension was kept at 6.00 × 0.45 mm and the scanning speed was 100 mm/s.

### 2.4. LC–ESI–Orbitrap–MS Analysis

To characterize the main metabolites representative of each sample, the LC–ESI–Orbitrap–MS method was developed. Each extract was dissolved 1:100 with methanol and a 10 μL aliquot injected into the analytical system. A duplicate of each sample was carried out, obtaining a total of 36 analyzed samples. Experiments were run using a Thermo Scientific liquid chromatography system, equipped with a quaternary Accela 600 pump and an Accela auto sampler, in conjunction with a linear Trap–Orbitrap hybrid mass spectrometer (LTQ–Orbitrap XL, Thermo Fisher Scientific, Bremen, Germany), combining a linear trap quadrupole (LTQ) and an Orbitrap mass analyzer with an electrospray ionization (ESI) source. Chromatographic separation was obtained using an X-Select T3 C18 reversed phase column (2.1 × 150 mm, 3.5 µm particle size) (Waters, Milford, Massachusetts). The mobile phase consisted of solvent A (water + 0.1% formic acid) and solvent B (acetonitrile + 0.1% formic acid). A linear gradient program at a flow rate of 0.200 mL/min was used: 0–3 min, from 0 to 10% (B); 3–25 min, from 10 to 20% (B); 25–35 min, from 20 to 30% (B); 35–40 min, from 30 to 60% (B); 40–45 min, from 60 to 100% (B); then 0% (B) for 5 min. The mass spectrometer was operated in negative ion mode. The ESI source parameters were the following: The capillary voltage −48 V; tube lens voltage −176.47 V; capillary temperature 300 °C; Sheath and Auxiliary Gas flow (N2) 15 and 5 (arbitrary units); Sweep gas 0 (arbitrary units); Spray voltage 3.50 V. MS spectra were acquired by full range acquisition covering *m*/*z* 200–1200 (Resolution: 30,000). For MS/MS experiments, a data-dependent scan experiment was established, with the selection of precursor ions corresponding to the most intense peaks observed in the previous LC–MS analysis (threshold value 300). 

Compounds were identified on the basis of their spectral characteristic fragmentation and retention time, with comparison with data reported in literature and databases. Data acquisition, data analysis, and instrument control were performed by Xcalibur software version 2.1 (Thermo Scientific™, Waltham, MA, USA)

### 2.5. PCA

Principal component analysis (PCA) is a multivariate data analysis technique used to reveal important patterns correlating to physiological, genetic, and environmental issues and has been used widely in assessing the differences between plant varieties at a metabolomics level [25]. An m × n matrix (where m is the number of samples and n is the number of variables) was used in PCA analysis of data obtained from HPTLC. For matrix building, the variables were taken from the pseudo-chromatogram and reported as the area % corresponding to intensities of the individual retention time factors of the most intense spots in the fingerprint of each sample. PCA was performed on the dataset scaled by unit variance with the Factor MineR package of R 2.15.2 software (R Foundation for Statistical Computing, Vienna, Austria). The results of the analysis are presented in terms of score and loading plots. 

Similarly, an m × n matrix (where m is the number of samples and n is the number of variables) was used in PCA analysis of data obtained from LC–ESI–Orbitrap–MS. The untargeted approach was obtained working with the base peak chromatograms derived from LC–MS (negative ion mode), which were evaluated using a platform independent open source software package called MZmine (http://mzmine.sourceforge.net/). By means of this toolbox normalization by total raw signal and excluding noise from LC–MS profiles (Noise level 5.0 E3—all data points below this intensity level were ignored), 280 peaks were detected and the peak area was determined [26].

After transferring the processed data in tabular format (cvs file), further analysis of the data matrix (36 observation and 280 variables) were made by SIMCA (+) software 12.0 (Umetrix AB, Umea, Sweden) by PCA. PCA was achieved by measuring the peak areas obtained from LC/MS analysis [27]. Pareto scaling was applied to data before multivariate data analysis. 

### 2.6. Antiradical Activity by Diphenyl-1-Picarylhydrazyl (DPPH) and TEAC Assays

The radical scavenging activity assay was performed according to the method proposed by Brand–Williams (1995) [28] with some modifications. 

The ABTS free radical scavenging activity of each sample was determined according to the method described by Petretto et al. (2015) [29].

### 2.7. Determination of Total Phenols

Total phenols were estimated by a colorimetric assay based on procedures described by Lizcano et al. (2010) [30] and by Maldini et al., 2016 [16]. 

### 2.8. Statistical Analysis

All experiments were carried out in triplicate. Statistical analyses were performed by comparison of ethanolic extracts from red fruits, yellow fruits, and leaves of *Arbutus unedo* collected in different areas of Sardinia, with unpaired Student’s *t*-test using Sigma-Stat v. 3.5 software (Systat Software GmbH, Erkrath, Germany). The distribution of samples was performed by the Kolmogorov–Smirnov and Shapiro tests. The strength of association between variables was investigated with the Pearson product moment correlation coefficient (data normally distributed). A *p* ≤ 0.05 was considered statistically significant.

## 3. Results and Discussion

### 3.1. HPTLC–PCA Analysis

The power of HPTLC analysis is to get characteristic fingerprints of secondary metabolites occurring in numerous biological samples (~20) in a single run. Thus, it was here employed to quickly compare *A. unedo* samples (red fruit, yellow fruit, and leaves) from different places of collection.

**Figure 1:**
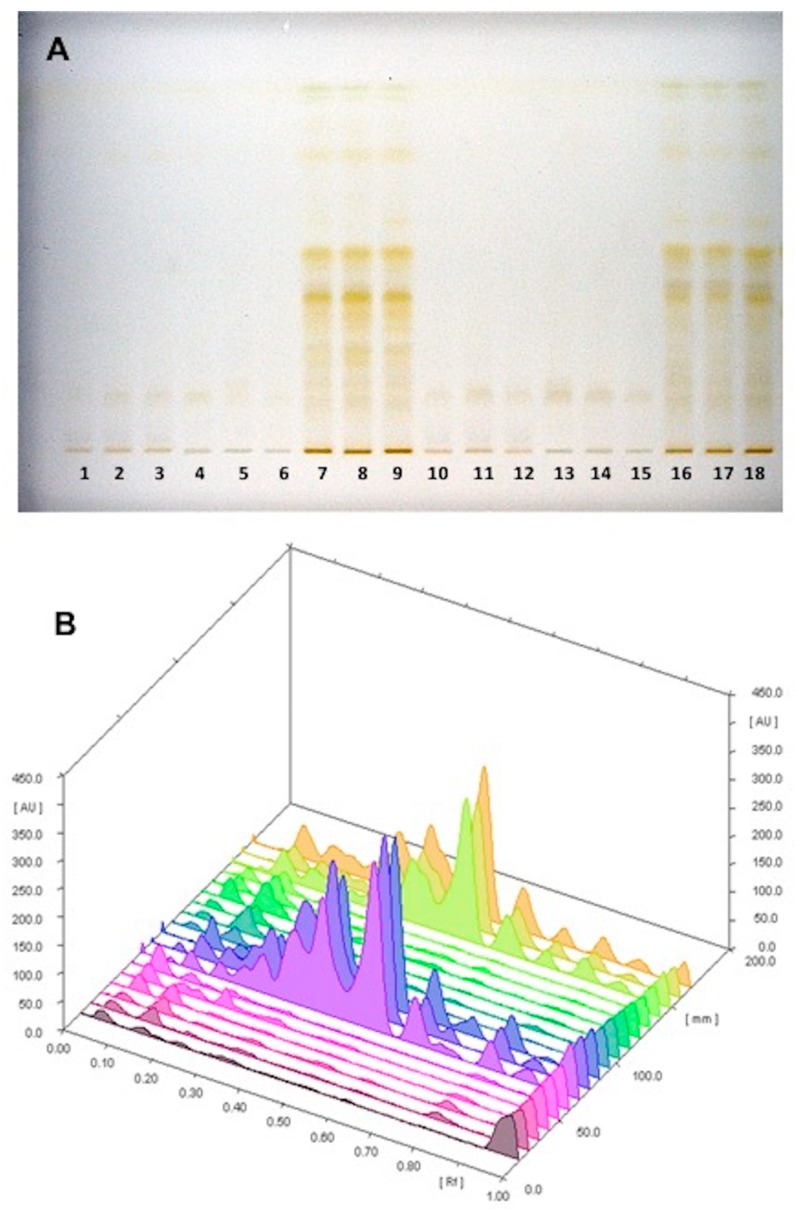
High Performance Thin Layer Chromatography (HPTLC) fingerprint (**A**) and three-dimensional (3D) densitograms (**B**) of *Arbutus unedo* samples (red fruit, yellow fruit, and leaves) from different places of collection (Sassari (SS) and archipelago of La Maddalena (LM)). 1. Red fruit LM 1; 2. Red fruit LM 2; 3. Red fruit LM 3; 4. Yellow fruit LM 1; 5. Yellow fruit LM 2; 6. Yellow fruit LM 3; 7. Leaves LM 1; 8. Leaves LM 2; 9. Leaves LM 3; 10. Red fruit SS 1; 11. Red fruit SS 2; 12. Red fruit SS 3; 13. Yellow fruit SS 1; 14. Yellow fruit SS 2; 15. Yellow fruit SS 3; 16. Leaves SS 1; 17. Leaves SS 2; 18. Leaves SS 3.

**Figure 2:**
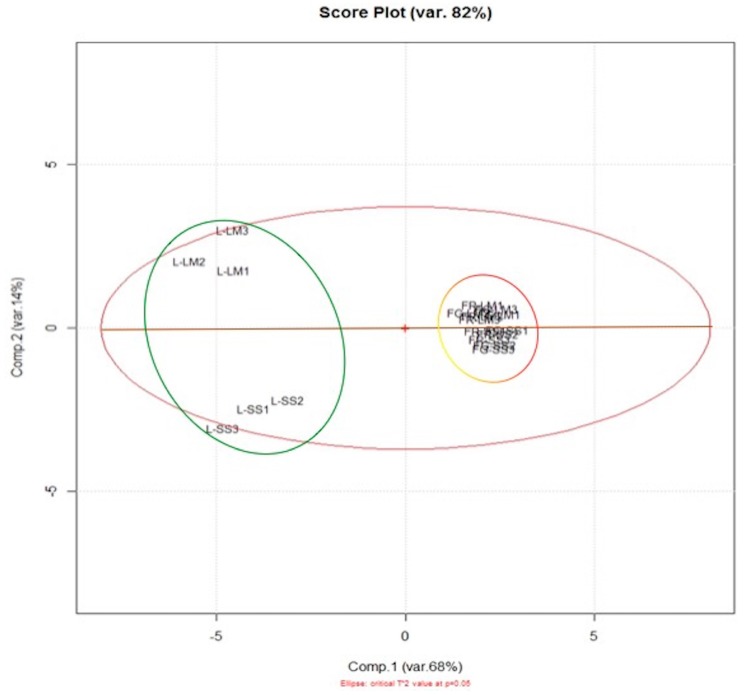
Score plot obtained from HPTLC data. (L = Leaves; FR = Red Fruit; FG = Yellow Fruit; SS = Sassari; LM = La Maddalena).

By using an optimized separation method, the different pigments are highlighted, using UV light at 254 or 366 nm or under reflectance and transmission white light (WRT Figure 1A). HPTLC metabolomics patterns didn’t show qualitative differences among samples collected in Sassari or in La Maddalena (Figure 1A). Successively, densitometric analysis was carried out at 254 nm and 366 nm. The HPTLC 3D chromatogram recorded at 254 nm in Figure 1B shows the characteristic traces of *A. unedo* red fruit, yellow fruit, and leaves from La Maddalena (1–9) and Sassari (10–18). Densitometer scanning at 254 nm wavelength was the most useful to characterize and differentiate extracts; thus, Rf values with a corresponding area for each spot were obtained and designated to produce an m × n matrix made up by 18 observations and 16 variables (metabolites detected). A PCA was conducted on the dataset to provide an overview to discriminate samples on the basis of the tissue and the geographical origin. Figure 2 shows the Score Plot obtained for a studied data set. The first principal component explains 68% of the variance, while the second principal component explains the 14%. Along the first component, samples are separated into two groups: In the negative values is located the group relative to leaves and in the positive values, red and yellow fruit. Along the second component, samples are located on the basis of their origin. All samples from La Maddalena are placed at positive values, whilst samples from Sassari are at negative values.

### 3.2. LC–ESI–Orbitrap–MS Analysis and PCA

**Figure 3:**
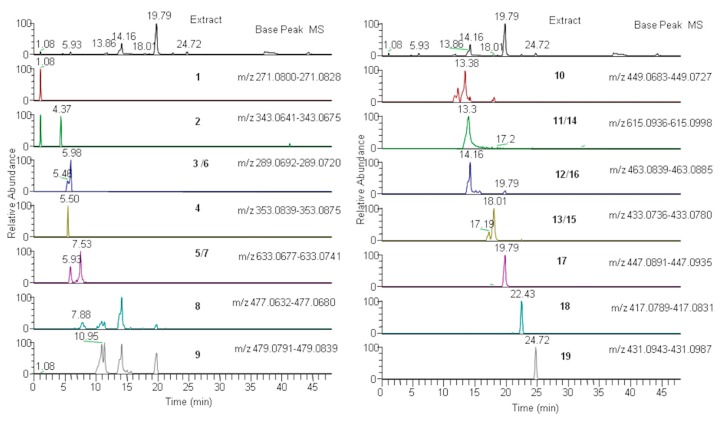
LC–ESI–MS/MS (Liquid Cromatography Elettrospray Ionization Mass (Mass) Spectrometry) profile and reconstructed ion chromatograms of *Arbutus* leaves extract. Number corresponds to the identified metabolites reported in Table 1.

With the aim to identify which secondary metabolites are responsible with a major impact for the variation of the analyzed samples, metabolites profiling of *A. unedo* leaves and red and yellow fruit ethanolic extracts from La Maddalena and Sassari were analyzed by liquid chromatography, coupled with high resolution mass spectrometry. Nineteen metabolites were detected and putatively identified (Table 1) according to the information obtained from accurate mass fragmentation spectra and then the obtained results were confirmed with those present in the literature and database. All the identified compounds were already reported in *A. unedo*. The main detected metabolites belong to flavonoids, principally quercetin, kaempferol, and myricetin derivatives (9–19) [31,32,33,34,35]. Quercetin and kaempferol derivatives were both similarly detected in leaves and fruit of *A. unedo* from Sassari and La Maddalena area, while myricetin derivatives are differently distributed in the samples; in fact, myricetin hexoside (9) was detected only in leaves. Other identified compounds belong to catechin and epicatechins (3 and 5) and galloyl organic acid derivative groups (2 and 8) [31] that, in the present study, were found both in fruit and leaves of the analyzed samples. In addition, other secondary metabolites belonging to ellagitannins class (4 and 7) were detected. These were found in all analysed samples, with the exception of yellow fruit of La Maddalena (LMFG) and red fruit of Sassari (SSFR). Finally, chlorogenic acid (6), present in all analysed samples, and arbutin (1), detected in all the samples except in yellow fruit from Sassari, were also identified. Figure 3 shows LC–HRMS and extracted ion chromatograms of compounds 1–19 identified in La Maddalena leaves extract.

The collected LC–ESI–Orbitrap–MS data were then processed by multivariate data analysis. With the aim to perform a comparative study of *Arbutus* extracts, the PCA approach was applied in an untargeted fashion to acquired raw data. LC/MS chromatograms were then pre-processed with the software MZmine to compensate for differences in retention time and *m*/*z* between the chromatographic runs. The pre-processed chromatograms were transferred as a peak list table, with rows corresponding to the individual samples, and columns corresponding to the integrated and normalized peak areas.

**Figure 4:**
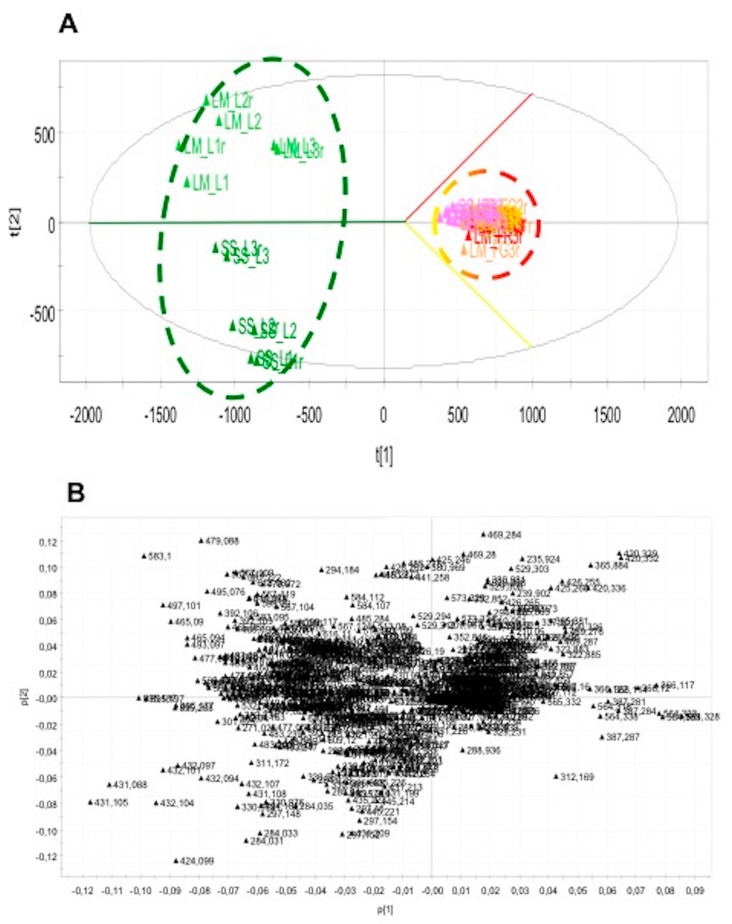
Score plot (**A**) and loading plot (**B**) obtained from HR–LCMS data. (L = Leaves; FR = Red Fruit; FG = Yellow Fruit; SS = Sassari; LM = La Maddalena).

PCA was developed by working with the peak areas of the total peaks present in the LC/MS dataset (excluding the noisy) in such a way that a matrix was obtained considering these areas, each corresponding to specific *m*/*z* values (variables), and the column of the matrix was realized by the different analyzed samples. Score scatter and loading plots are reported in Figure 4A,B, respectively (LC–ESI–Orbitrap–MS data obtained in negative analysis). The first component explained the 50% of variance while the second explained the 8.9%. The legend of classification can be read as follows: LMFG (Yellow Fruit La Maddalena); LMFR (Red Fruit La Maddalena); LML (Leaves La Maddalena); SSFG (Yellow Fruit Sassari); SSFR (Red Fruit Sassari); SSL (Leaves Sassari). The score plot shows that HR–LC–MS–PCA discriminates samples on the basis of the tissue and of their geographical area, and confirms data obtained in previous analysis, apart from yellow and red fruit where geographical discrimination is less clear than that obtained by HPTLC–PCA.

### 3.3. Antioxidant Activity

Two different assays were employed to assess the antioxidant activities of different *A. unedo* extracts: (a) DPPH assay, a direct method based on the scavenging of the radical; and (b) ABTS assay, based on the inhibition of cation radical by antioxidants. IC50 value (the quantity of an extract able to neutralize the 50% of the radical) was taken into account to evaluate the antioxidant activity because the measurement of the absolute value for the antioxidant activity of an extract is contingent on many variables, comprising degradation during the analysis and matrix interference, and these contribute to the possibility of a wrong value. Table 2 reports the results obtained for both DPPH and ABTS assays. Regarding the DPPH assay, leaves extracts of both Sardinia’s areas (Sassari and La Maddalena) showed an antioxidant activity significantly higher than red fruits and yellow fruits extracts, respectively, at time zero and after 30 min, a time when the reaction was stable and complete.

Data were expressed as means ± SD. A *p* < 0.05 was considered significant. FR SS = Red fruits of *A. unedo* from Sassari; FG SS = Yellow fruits of *A. unedo* from Sassari; L SS = Leaves of *A. unedo* from Sassari; FR LM = Red fruits of *A. unedo* from La Maddalena; FG LM = Yellow fruits of *A. unedo* from La Maddalena; L LM = Leaves of *A. unedo* from La Maddalena.

La Maddalena leaves extracts presented a higher free radical scavenging effect than those from Sassari, even if not statistically significantly different. Similarity, the ABTS results were in agreement with those obtained for DPPH, even if ABTS gave the best results, showing for Sassari and La Maddalena leaves extracts IC50 values of 2.35 ± 0.06 and 2.72 ± 1.68 µg/mL, respectively, lower than those obtained for Trolox, reference compound.

The best results showed by ABTS assay over DPPH depend on the fact that ABTS can be used over a wider range of pH and is able to measure the antioxidant capacity of both water-soluble and lipid-soluble metabolites, since it can be dissolved in both aqueous and organic media, unlike DPPH, which can only be solubilized in alcoholic media [36].

Determination of total phenolic content was carried out for all extracts using Folin–Ciocalteu assay (Table 3). Obtained results were in agreement with the demonstrated antioxidant activity of the three ethanolic extracts obtained from samples from different areas of Sardinia.

Data were expressed as means ± SD of three independent experiments. Each result of red and yellow fruits showed a positive correlation with DPPH (* *p* < 0.01) and ABTS results († *p* < 0.05).

However, a positive Pearson correlation between antioxidant activity measured with DPPH and ABTS and total phenolic contents was detected only in red and yellow fruits, probably because in leaves, antioxidant activity measured with DPPH and ABTS reached saturation early as low concentrations, due to the occurrence of different compounds in leaves extracts. Regardless, the higher amount of total phenols of leaves as well as red fruits and yellow fruits demonstrated that antioxidant activity is governed by phenolic amounts, confirming previous data [37]. 

## 4. Conclusions

In conclusion, in a totally untargeted approach, the power of HPTLC in discriminating origin and organs of samples was evaluated. The obtained results were compared with a more performant as well as more complex technique, mainly HRMS. Comparing the results obtained for both the techniques and visualized by PCA, the discrimination results are comparable. Then, for a preliminary untargeted approach, by integrating HPTLC with PCA, it’s possible to discriminate samples with good confidence and in a short time.

The second aim of the paper was to carry out a qualitative analysis of the main compounds occurring in the samples from different origins and organs. This objective was reached by data collected by LC–HRMS, which permitted it to assign an elemental formula for the main *m*/*z* value and LC–HR–MS (MS) to enrich information with a fragmentation pattern useful to complete identification of compounds.

Finally, we also confirm that the high antioxidant activity is related to the amount of total phenolic content. 

The present study highlighted that the qualitative and quantitative chemical diversity detected by analytical techniques, related to the slightly different habitats in which *Arbutus unedo* grow, give rise, after multivariate analysis, to a discrimination of the studied samples. HPTLC and LC–ESI–Orbitrap–MS provide different and complementary data and together these may be employed to really differentiate between a wide variety of crude drug powders and herbal medicinal products.

## Figures and Tables

**Table 1:** Metabolites identified in *A. unedo* leaves and fruit extracts by LC–ESI/LTQOrbitrap/MS and LC–ESI/LTQOrbitrap/MS/MS analysis. (x = present; nd = not detected).

No.	RT	[M − H]^−^	Molecular Formula	Δ PPM	MS/MS	Identity	LMFG	LMFR	LML	SSFG	SSFR	SSL
1	1.08	271.0814	C_12_H_15_O_7_	0.6	139.08	arbutin	x	x	x	nd	x	x
2	4.3	343.0658	C_14_H_15_O_10_	−0.6	283.8/191.1	galloyl quinic acid	x	x	x	x	x	x
3	5.4	289.0708	C_27_H_21_O_18_	−1.5	275.9/245.1/205.1/144.2	catechin/epicatechin	x	x	x	x	x	x
4	5.5	353.0857	C_16_H_17_O_9_	−2.8	262.8/191.01/171.7	chlorogenic acid	nd	nd	x	nd	nd	x
5	5.8	633.0712	C_27_H_21_O_18_	−1.5	463.06/300.9	strictinin ellagitannin	nd	x	x	x	nd	x
6	5.9	289.0706	C_27_H_21_O_18_	−1.5	275.9/245.1/205.1/144.2	catechin/epicatechin	x	x	x	x	x	x
7	7.6	633.0709	C_27_H_21_O_18_	−1.5	463.06/300.9	strictinin ellagitannin	nd	x	x	x	nd	x
8	8.0	477.0656	C_21_H_17_O_13_	0.2	462.0/366.07/325.05/191.03/164.7	digalloylquinic shikimic acid	x	x	x	x	nd	x
9	10.9	479.0815	C_21_H_19_O_13_	−1.43	451.2/354.7/316.02/297.6/165.8/145.5	myricetin glucoside	nd	nd	x	nd	nd	x
10	13.0	449.0705	C_20_H_17_O_12_	−1.7	317.02/183.3/149.5	myricetin pentoside	nd	x	x	nd	nd	x
11	13.3	615.0967	C_28_H_23_O_16_	1.8	463.08/301.03	quercetin galloylhexoside isomer	x	x	x	x	x	x
12	14.2	463.0862	C_21_H_19_O_12_	−1.5	316.02/284.8/183.5	myricetin rhamnoside	nd	x	x	x	nd	x
13	17.1	433.0758	C_20_H_17_O_11_	−1.12	355.3/312.7/301.03/283.8/216.2	quercetin pentoside	x	x	x	x	x	x
14	17.2	615.0960	C_28_H_23_O_16_	1.02	463.08/301.03/265.3/241.08	quercetin galloylhexoside isomer	x	x	x	x	x	x
15	17.7	433.0758	C_20_H_17_O_11_	−1.1	301.03/265.8/247.7/219.2/181.9/134.9	quercetin pentoside	x	x	x	x	x	x
16	19.5	463.0860	C_21_H_19_O_12_	0.8	404.3/316.02/301.04/282; 2	isoquercitrin	nd	x	x	x	nd	x
17	19.7	447,0913	C_21_H_19_O_11_	−1.2	301.03/287.9/178.9	quercitrin	x	x	x	x	x	x
18	22.4	417.0810	C_20_H_17_O_10_	−0.9	374.9/285.04/175.6	kaempferol pentoside	nd	x	x	x	x	x
19	24.7	431.0965	C_21_H_19_O_10_	−1.09	285.03/235.2/195.9	Kaempferol-rhamnoside (afzelin)	x	x	x	nd	x	x

(L = Leaves; FR = Red Fruit; FG = Yellow Fruit; SS = Sassari; LM = La Maddalena). Compounds were putatively identified with ID level (Identification level): Putatively annotated compounds (e.g., without chemical reference standards, based upon physicochemical properties and/or spectral similarity with public/commercial spectral libraries).

**Table 2:** Scavenging of 50% of DPPH and ABTS radical by Trolox and ethanolic extracts of *Arbutus unedo* of different areas of Sardinia, at different time points.

	DPPH IC_50_			ABTS IC_50_		
	0 min	30 min	*p* Value	0 min	50 min	*p* Value
**Trolox** (µg/mL)	13.50	6.15		3.34	3.28	
***A. unedo* Sassari Red fruits** (µg/mL)	229.09 ± 81.75	103.59 ± 45.97	**Time 0 min** LSS vs. FR SS ***p* < 0.05** LSS vs. FG SS ***p* < 0.05**	52.71 ± 16.13	21.43 ± 7.34	**Time 0 min** LSS vs. FR SS ***p* < 0.01** LSS vs. FG SS ***p* < 0.05**
***A. unedo* Sassari Yellow fruits** (µg/mL)	300.92 ± 107.31	131.53 ± 44.61		101.11 ± 53.44	40.33 ± 21.95	
***A. unedo* Sassari leaves** (µg/mL)	29.37 ± 5.87	15.18 ± 4.11	**Time 30 min** LSS vs. FR SS ***p* < 0.05** LSS vs. FG SS ***p* < 0.01**	7.63 ± 1.38	2.35 ± 0.06	**Time 50 min** LSS vs. FR SS ***p* < 0.01** LSS vs. FG SS ***p* < 0.05**
***A. unedo* La Maddalena Red fruits** (µg/mL)	235.67 ± 43.72	116.84 ± 16.54	**Time 0 min** L LM vs. FR LM ***p* < 0.01** L LM vs. FG LM ***p* < 0.01**	79.36 ± 46.09	34.91 ± 23.07	**Time 0 min** L LM vs. FG LM ***p* < 0.05**
***A. unedo* La Maddalena Yellow fruits** (µg/mL)	380.14 ± 90.72	180.30 ± 75.48		96.09 ± 40.98	49.89 ± 17.62	
***A. unedo* La Maddalena leaves** (µg/mL)	23.35 ± 4.25	8.85 ± 7.79	**Time 30 min** L LM vs. FR LM ***p* < 0.01** L LM vs. FG LM ***p* < 0.05**	6.73 ± 2.14	2.72 ± 1.68	**Time 50 min** L LM vs. FG LM ***p* < 0.01**

**Table 3:** Determination of phenols by Folin–Ciocalteu’s method.

Extract Concent Ration (µg/mL)	GAE *A. Unedo* Sassari Red Fruits (µg GAE)	GAE *A. Unedo* Sassari Yellow Fruits (µg GAE)	GAE *A. Unedo* Sassari Leaves (µg GAE)	GAE *A. Unedo* La Maddalena Red Fruits (µg GAE)	GAE *A. Unedo* La Maddalena Yellow Fruits (µg GAE)	GAE *A. Unedo* La Maddalena Leaves(µg GAE)	Pearson Product Moment Correlation
100	67.10 ± 17.14 *^†^	43.62 ± 11.85 *^†^	347.56 ± 126.05	53.16 ± 35.75 *^†^	50.62 ± 20.64 *^†^	327.90 ± 126.75	* vs. DPPH *p* < 0.01 ^†^ vs. ABTS *p* < 0.05
50	35.33 ± 9.37 *^†^	22.72 ± 6.41 *^†^	179.52 ± 70.68	26.42 ± 11.73 *^†^	26.42 ± 8.04 *^†^	181.55 ± 84.65	
25	23.67 ± 4.29 *^†^	16.13 ± 9.12 *^†^	116.46 ± 36.12	21.11 ± 11.83 *^†^	18.10 ± 5.64 *^†^	143.84 ± 67.03	
10	10.15 ± 3.27 *^†^	6.78 ± 2.43 *^†^	51.50 ± 15.93	9.53 ± 3.44 *^†^	8.30 ± 2.42 *^†^	64.56 ± 33.28	
5	6.63 ± 1.16 *^†^	4.24 ± 0.55 *^†^	29.01 ± 4.24	6.17 ± 3.36 *^†^	5.04 ± 3.89 *^†^	38.12 ± 17.86	
1	3.17 ± 1.66 *^†^	2.40 ± 0.70 *^†^	10.04 ± 1.54	4.91 ± 1.69 *^†^	2.52 ± 1.17 *^†^	18.54 ± 14.44	

* *p* < 0.01, † *p* < 0.05.
